# A genetic system for *Citrus Tristeza Virus* using the non-natural host *Nicotiana benthamiana*: an update

**DOI:** 10.3389/fmicb.2013.00165

**Published:** 2013-07-02

**Authors:** Silvia Ambrós, Susana Ruiz-Ruiz, Leandro Peña, Pedro Moreno

**Affiliations:** ^1^Centro de Protección Vegetal y Biotecnología, Instituto Valenciano de Investigaciones AgrariasMoncada, Valencia, Spain; ^2^Ciudad Politécnica de la Investigación, Instituto de Biología Molecular y Celular de Plantas (UPV-CSIC)Valencia, Spain

**Keywords:** CTV, infectious cDNA clones, agroinoculation, *Agrobacterium tumefaciens*, oncogenic strains, graft transmission, *N. benthamiana* protoplasts, RNA silencing suppressor

## Abstract

In nature *Citrus tristeza virus* (CTV), genus *Closterovirus*, infects only the phloem cells of species of *Citrus* and related genera. Finding that the CTV T36 strain replicated in *Nicotiana benthamiana* (NB) protoplasts and produced normal virions allowed development of the first genetic system based on protoplast transfection with RNA transcribed from a full-genome cDNA clone, a laborious and uncertain system requiring several months for each experiment. We developed a more efficient system based on agroinfiltration of NB leaves with CTV-T36-based binary plasmids, which caused systemic infection in this non-natural host within a few weeks yielding in the upper leaves enough CTV virions to readily infect citrus by slash inoculation. Stem agroinoculation of citrus and NB plants with oncogenic strains of *Agrobacterium tumefaciens* carrying a CTV-T36 binary vector with a GUS marker, induced GUS positive galls in both species. However, while most NB tumors were CTV positive and many plants became systemically infected, no coat protein or viral RNA was detected in citrus tumors, even though CTV cDNA was readily detected by PCR in the same galls. This finding suggests (1) strong silencing or CTV RNA processing in transformed cells impairing infection progress, and (2) the need for using NB as an intermediate host in the genetic system. To maintain CTV-T36 in NB or assay other CTV genotypes in this host, we also tried to graft-transmit the virus from infected to healthy NB, or to mechanically inoculate NB leaves with virion extracts. While these trials were mostly unsuccessful on non-treated NB plants, agroinfiltration with silencing suppressors enabled for the first time infecting NB plants by side-grafting and by mechanical inoculation with virions, indicating that previous failure to infect NB was likely due to virus silencing in early infection steps. Using NB as a CTV host provides new possibilities to study virus-host interactions with a simple and reliable system.

## Introduction

*Citrus tristeza virus* (CTV), a member of genus *Closterovirus*, is one of the more economically important plant viruses. Almost 100 million trees propagated on sour orange (*Citrus aurantium* L.) rootstocks died worldwide from different tristeza epidemics, and presently, many millions more propagated on decline-tolerant rootstocks are debilitated by severe CTV isolates inducing stem pitting in commercial citrus varieties regardless of the rootstock used (Bar-Joseph and Dawson, 2008; Moreno and Garnsey, 2010).

CTV virions (2000 × 10–12 nm) are composed of a single-stranded, positive-sense genomic RNA (gRNA) of about 20 kb and two coat proteins of 25 (CP) and 27 (CPm) kDa that encapsidate about 97 and 3% of the gRNA, respectively (Bar-Joseph and Lee, 1989; Satyanarayana et al., 2004; Gowda et al., 2009). The CTV gRNA has 12 open reading frames (ORF) and untranslated regions (UTR) of 107 and 273 nt at the 5′ and 3′ termini, respectively (Karasev et al., 1995). ORFs 1a and 1b, encompassing the 5′ half of the genome, encode replicase-related proteins that are translated from the gRNA and contain papain-like protease, methyltransferase-like, helicase-like and RNA-dependent RNA polymerase domains. The ten 3′- proximal ORFs encode proteins p33, p6, p65, p61, p27, p25, p18, p13, p20, and p23, which are expressed via 3′ coterminal subgenomic RNAs (sgRNAs) (Hilf et al., 1995), promoted by internal controller elements (Gowda et al., 2001). Proteins p6, p65, p61, p27, and p25 are part of a module conserved among closteroviruses that is involved in virion assembly and movement (Satyanarayana et al., 2000, 2004; Dolja et al., 2006; Gowda et al., 2009; Tatineni et al., 2010). The p33, p18, and p13 proteins are dispensable to systemically infect some citrus species (Tatineni et al., 2008), but they are required to invade others like grapefruit (*C. paradisi* Macf.) and sour orange (Tatineni et al., 2011). Moreover, p33 is required in CTV-infected plants to exclude superinfection by isolates of the same strain (Folimonova et al., 2010; Folimonova, 2012), and the expression ratio between p33 and p13 or p18 seems to determine the stem pitting symptom (Tatineni and Dawson, 2012). Proteins p25, p20, and p23 have been shown to act as silencing suppressors in *Nicotiana benthamiana* (NB) and *N. tabaccum* plants (Lu et al., 2004), with p23 being also a pathogenicity determinant (Ghorbel et al., 2001; Fagoaga et al., 2005; Albiach-Martí et al., 2010).

In nature, the CTV host range is restricted to species of a few genera within the subfamily *Aurantioideae*, and within infected plants, the virus invades only phloem tissues. Although CTV was experimentally transmitted to *Passiflora gracilis* and *P. caerulea* (Müller et al., 1974; Roistacher and Bar-Joseph, 1987), two perennial vines, trials to mechanically transmit it to herbaceous and other non-rutaceous woody species, including NB and other *Nicotiana* species, were unsuccessful (Müller and Garnsey, 1984; our unpublished results). Moreover, although citrus can be mechanically inoculated by slashing citrus stems with CTV virions (Garnsey et al., 1977), all attempts to mechanically inoculate them with virion RNA or RNA transcripts from a cDNA clone were unsuccessful (Satyanarayana et al., 2001). These limitations and the large size of the CTV genome that hindered preparation of full-length cDNA clones and of intact RNA transcripts for inoculation greatly delayed development of a genetic system for this virus. After Navas-Castillo et al. (1997) showed that CTV virions were able to replicate in NB protoplasts, Satyanarayana and associates (1999) developed a full-length cDNA clone of the CTV isolate T36 (CTV-T36), from which they synthesized *in vitro* RNA transcripts that infected NB protoplasts and produced normal CTV virions. Due to the large size and fragility of the RNA transcripts and the difficulty to inoculate protoplasts with such large RNAs, the protoplast infection rate was only about 10^−4^ and the amount of virions produced was insufficient to infect citrus plants by mechanical inoculation. Virion amplification by successive cycles of protoplast inoculations yielded amounts of virions which were able to systemically infect citrus plants and incite the symptoms characteristic of the wild T36 isolate (Satyanarayana et al., 2001). However, the above limitations and frequent failures in virion amplification and transfer between protoplast batches made this genetic system very tenuous.

To overcome these problems we developed an improved genetic system based on agroinfiltration of NB leaves with binary plasmids carrying a cDNA of the CTV-T36 genome and different silencing suppressors and performed a time-course analysis of CTV accumulation in those leaves (Ambrós et al., 2011). Unexpectedly, we found that agroinoculated plants of this species, presumed to be “non-host,” were systemically invaded by CTV-T36 with high viral titers, particularly in the upper leaves in which the virus eventually invaded some non-phloem tissues and incited typical disease symptoms. Citrus plants mechanically inoculated with virions produced in NB became systemically infected, displayed the symptoms characteristic of the wild CTV-T36 isolate, and restricted the virus to the phloem, suggesting that replication and movement in NB tissues does not alter CTV-T36 properties.

This new genetic system based on the use of NB as an intermediate host was simpler and more reliable than the former system based on protoplast transfection but it still had at least three potential limitations:

1. Direct agroinoculation of citrus plants would be an easier and faster procedure than using NB as an intermediate host for producing virions. Although previous efforts to agroinfect citrus with CTV using binary plasmids and different *A. tumefaciens* strains were unsuccessful (Gowda et al., 2005, and our unpublished data), virulence and transformation efficiency of *A. tumefaciens* strains are widely determined by experimental conditions and specific interactions with the plant that make some *Agrobacterium* strains more virulent than others in a particular host species, including members of the *Rutaceae* (Cervera et al., 1998). Transient and stable transformation of herbaceous and woody plants has been performed mainly with non-oncogenic (disarmed) strains in which the oncogenes of the wild-type T-DNAs were removed (Gelvin, 2005). In this work we tried agroinoculation of citrus plants using two oncogenic strains, the virulent strain C58 (pTiC58) and the supervirulent strain A281 (pTiBo542), transfected with binary vectors carrying a plant expression marker gene and a cDNA of CTV-T36.
2. The present genetic system relies on the ability of CTV-T36 to replicate in NB cells and to eventually move cell-to-cell and long distance, but the ability of other CTV genotypes to infect this host remains unknown. Therefore, testing if these virions can replicate in NB protoplasts is a preliminary step to develop a similar genetic system for other genotypes, a step that would be easier and faster if virions could be successfully introduced into NB cells. Here we tried to develop a procedure to mechanically inoculate NB plants with virions of different CTV isolates.
3. CTV-infected NB plants show dwarfing, necrosis and often die after a few months and further work with different CTV hybrid constructs or mutants, would require a procedure to maintain these constructs in NB without needing new agroinoculations. For this purpose here we developed a graft transmission procedure to transmit CTV from infected to healthy NB plants.

We found that the oncogenic *Agrobacterium* strains efficiently induced tumors expressing GUS in different plant species, including citrus, and that systemic CTV infection developed in some NB plants. However, CTV virions were not detected in tumors of citrus plants. Mechanical inoculation of CTV virions on NB plants agroinfiltrated previously with a silencing suppressor resulted in systemic infection with CTV-T36, but not with CTV T318A, even though this latter isolate replicated and accumulated in NB protoplasts to the same extent as CTV-T36. CTV-T36 was readily graft transmitted from infected to healthy NB plants after agroinfiltrating the receptor plants with a silencing suppressor. The possibilities and limitations of the new genetic system are further discussed.

## Materials and methods

### Virus isolates and plant growth

The CTV isolate 947R is a clonal virus population obtained from the infectious CTV-T36 cDNA clone from Florida (Satyanarayana et al., 2001) that is maintained in Mexican lime [*C. aurantifolia* (Chritm.) Swing.] and alemow plants (*C. macrophylla* West.). Isolates T305 and T318A, inducing stem pitting on sweet orange [*C. sinesis* (L.) Osb.] and grapefruit (Ruiz-Ruiz et al., 2006; Sambade et al., 2007), and T385, a very mild isolate (Vives et al., 1999), are part of the IVIA collection of citrus viruses.

Citrus plants were grown in individual pots as previously reported (Arregui et al., 1982) and kept in a temperature-controlled (18/26°C night/day) greenhouse, whereas *N. benthamiana* plants were grown in a customized growth chamber kept at 23–24°C constant temperature and 50–60% relative humidity with a 16/8 h light/dark photoperiod.

### Binary vectors and Agrobacterium strains

**Figure 1:**
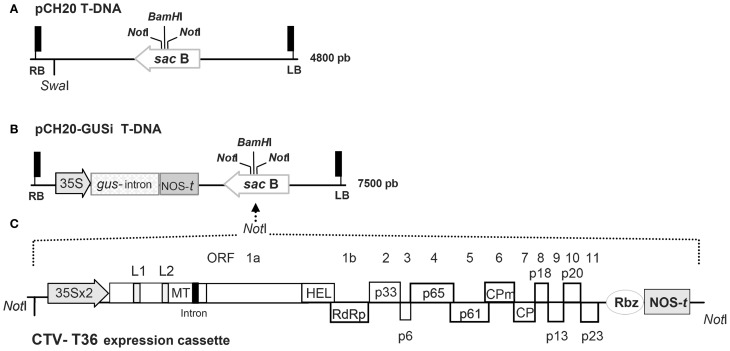
**Outline of the binary plasmids used in this study. (A)** Detail of the T-DNA region in the pCH20 binary vector with its size indicated at the right. RB and LB are the right and left borders, the *Not*I restriction sites for subcloning, the unique *Swa*I site of the T-DNA and the unique *Bam*HI site present in the *sac*B gene are indicated. **(B)** Detail of the T-DNA region in the pCH20-GUSi binary vector. The cassette of the *gus*-intron selectable marker gene containing a catalase intron is shown. Shadowed boxes represent the 35S promoter of the *Cauliflower mosaic virus* (CaMV) and the nopaline synthase terminator (NOS-*t*) present in the cassette. Other details as in **(A)**. **(C)** Schematic representation and relative orientation of the CTV-T36 expression cassette containing the full-genome cDNA clone CTV9R from the agroinfectious BAC CTV-vector (Ambrós et al., 2011). Shadowed boxes represent the double enhanced 35S promoter (35S × 2), the *Hepatitis delta virus* ribozyme (Rbz) and the NOS-*t* present in the cassette. The genome organization of the CTV gRNA is denoted by boxes indicating the open reading frames (ORFs) and their corresponding translation products. L1 and L2 mean the two leader papain-like proteases; MT, methyltransferase; HEL, helicase; RdRp, RNA-dependent RNA polymerase; CPm and CP, the minor and major coat proteins; intron, the *ST-LS1* intron incorporated in some CTV clones (Ambrós et al., 2011). The *Not*I sites flanking the construct are indicated and the strategy for subcloning into the T-DNA of pCH20-GUSi is marked with dotted arrow lines.

The BAC plasmid pCH20 (18 kb) contains two *Not*I sites flanking the unique *Bam*HI cloning site in the *sac*B gene of the T-DNA, a unique *Swa*I site and a selectable marker for resistance to kanamycin in bacteria (Hamilton, 1997) (Figure 1A). The *gus*-intron gene cassette from the pCAMBIA 2301 plasmid was PCR amplified using specific primers and the *Pfu* DNA polymerase (Stratagene). After phosphorylation, the ~2.7 kb amplified fragment was cloned into the unique *Swa*I site of pCH20 to obtain the pCH20-GUSi plasmid (20.8 kb) (Figure 1B). The catalase intron of the GUS marker gene blocks its expression in transformed *A. tumefaciens* ensuring that glucuronidase activity is possible only in eukaryotic cells. The CTV9R expression cassettes (with or without an intron in the ORF 1a) were *Not*I excised from the original BAC vectors (Ambrós et al., 2011) and ligated vía *Not*I to the pCH20-GUSi plasmid to yield the pCH20-GUSi-CTV vector. *E. coli* DH10B transformants of these plasmids were selected on LB plates with kanamycin (50 mg/l) and sucrose (5%), and purified plasmid used to transform *A. tumefaciens* strains by electroporation. The C58 (pTiC58) *A. tumefaciens* is an oncogenic wild-type strain from the nopaline group, and A281 (pTiBo542) is a transconjugant of C58 belonging to the L,L-succinamopine group (both maintained in the IVIA Collection of Plant Pathogenic Bacteria). Transformed cells of both strains were selected on Luria-Bertani medium (LB) containing rifampicin (25 mg/l) and carbenicillin (20 mg/l) for C58 or nalidixic acid (20 mg/l) for A281 strain. Plasmids transfected in these strains were additionally selected with kanamycin (50 mg/l).

### Stem and leaf agroinoculations

*A. tumefaciens* colonies of C58 and A281 strains harboring the pCH20-GUSi or pCH20-GUSi-CTV vector were grown overnight at 28°C in LB supplemented with appropriate antibiotics. After centrifugation, 20 μl of bacterial suspensions at OD600 1.0 were inoculated on the stems of the different plant hosts by wounding with a sterile scalpel in 3 (herbaceous hosts) or 5 (citrus) sites, and then protecting inoculation sites from desiccation with plastic wraps. Control plants were inoculated with water or *Agrobacterium* strains without binary vector. Tumor formation was monitored visually and expression analyses were performed at 3–5 weeks post-inoculation.

Agroinfiltration of NB or citrus leaves was performed as described (Ambrós et al., 2011), but in co-infiltration experiments, *A*. *tumefaciens* cultures harboring the candidate binary plasmids (empty or carrying the CTV genomic sequence) and those expressing the silencing suppressor p19 of *Tomato bushy stunt virus* (TBSV) (Voinnet et al., 2003) or p23 (CTV) (Lu et al., 2004) were mixed in a 2:1 ratio prior to infiltration.

### GUS and ELISA assays

Thin slices from the stem tumors induced by *Agrobacterium* C58 and A281 in tomato, *Nicotiana spp*. and Mexican lime plants were assayed individually for GUS activity at 4–6 (herbaceous hosts) or 6–8 (citrus) weeks after bacterial inoculations. GUS assays were performed by overnight incubation of tissue slices at 37°C with a 2 mM X-Gluc solution as described by Peña et al. (2004b).

CTV infection was monitored by a double-antibody sandwich-ELISA protocol using monoclonal antibodies 3DF1 and 3CA5 (Vela et al., 1986).

### RNA and DNA extract preparation

Total RNA extracts (RNAt) were prepared from citrus, NB or *N. occidentalis* leaves or stem tumors, ground to powder with liquid nitrogen following a standard protocol (Ruiz-Ruiz et al., 2007) and then treated with RNase-free DNase (Ambion) before using it as template for RT-PCR techniques. Extracts enriched in double-stranded RNA (dsRNA) were obtained from CTV-infected citrus bark as reported previously (Moreno et al., 1990).

Genomic DNA (gDNA) extracts were prepared from a tissue pool including two slices from individual tumours in each plant according to Llop et al. (1999).

### PCR, RT-PCR, and QRT-PCR reactions

Conventional PCR reactions to monitor transformation events were performed using gDNA extracts from tomato or citrus tumor tissues and four sets of primers: the primer set PM111 (5′-ATGACGCACAATCCCACTATCCTTCGC-3′)/PM250 (5′-GAGTGACCGCATCGAAACGCAGC-3′) amplifies a 581-bp fragment of the GUS-intron cassette; the set PM175 (5′-GCAGTCTCAGAACGAGGTGGC-3′)/BB2-5′ (5′-GGAAGGAGCTGACTGGGTTGAAGGC-3′) amplifies a 1063-bp fragment spanning the p23 ORF of CTV and the 5′ BAC polylinker; the set PM214b (5′-TTTCTGGGCGAACAGGTTGAAT-3′)/BB2-3′ (5′-GAAGACATACATGACAAAAACGCTAGACGGC-3′) amplifies a 1,5-kb fragment including the 32 first nucleotides of the CTV 5′UTR and the 3′ BAC polylinker; and the set PM118/PM119 flanking the intron insertion point in the CTV ORF 1a amplifies a 622-bp fragment (intron-containing constructs) or a 433-bp fragment (intron-less templates). PCR conditions were essentially as reported (Ruiz-Ruiz et al., 2006).

Reverse transcription (RT) followed by PCR amplification to detect CTV gRNA was performed with primers PM118–PM119 and 1–2 μ g DNase-treated RNAt as described (Ruiz-Ruiz et al., 2006; Ambrós et al., 2011). Control reactions included the absence of reverse transcriptase for each sample and negative reactions using water instead of RNAt, or RNAt from healthy plants or from plants agroinoculated with an empty binary vector. Positive controls were run by using RNAt or dsRNA-rich extracts from CTV-infected citrus plants, or PCR amplification of plasmid DNA containing CTV cDNA with or without the intron.

Quantitative assays (qRT-PCR) and estimations of the absolute number of T36 gRNA copies/ng of RNAt were performed as reported (Ruiz-Ruiz et al., 2007), including similar positive and negative controls as in conventional RT-PCR reactions.

### Transfection of NB mesophyll protoplasts and northern blot analysis

Isolation of mesophyll protoplasts from *N. benthamiana* leaves and transfection mediated by polyethylene glycol was as reported (Navas-Castillo et al., 1997; Satyanarayana et al., 1999). Virions from CTV isolates T36, T385, T305, and T318A were used for transfections. Protoplasts were harvested at 1–5 days post inoculation (dpi) and used to obtain RNAt extracts to analyze viral progeny (gRNA and sgRNAs) accumulation by Northern blot hybridization (Satyanarayana et al., 1999) with a digoxigenin-labeled riboprobe specific for the 3′ terminal region of the T318A gRNA.

### Mechanical and graft inoculation of NB plants

Mechanical inoculation of young NB plants (~1.5 months old) were performed by rubbing on the surface of three carborundum-dusted leaves 20–40 μl of virion extract from a sucrose gradient or crude sap extract from an infected plant. For graft transmission, symptomatic young shoots, or leaf petioles were excised from CTV-infected NB plants and used for V-shaped side-grafting on the stem of adult receptor plants. The grafts were protected with parafilm which was removed after 8–10 days. Before mechanical or side-graft inoculation, 3–4 fully expanded leaves of each receptor plant were agroinfiltrated with binary plasmids expressing a silencing suppressor protein as reported (Ambrós et al., 2011), with bacteria concentration being adjusted to 0.2 OD_600_.

### Indexing in citrus indicator plants

Infectivity bioassays of CTV virions from systemically infected leaves of agroinoculated NB plants were performed by slash-inoculation on four alemow plants (Garnsey et al., 1977). Inoculum consisted of virions purified in a sucrose gradient (Satyanarayana et al., 2001) or crude sap extracts, as indicated. Controls consisted of a similar number of indicator plants inoculated with virion extracts from citrus plants infected with the 947R CTV isolate. CTV infection of new leaves was detected at 1–2 months post inoculation (mpi) by ELISA and by symptom observation.

## Results

### Agroinoculation of CTV with oncogenic *A. tumefaciens* strains produces virus-infected tumours and systemic infection in NB but not in mexican lime

**Figure 2:**
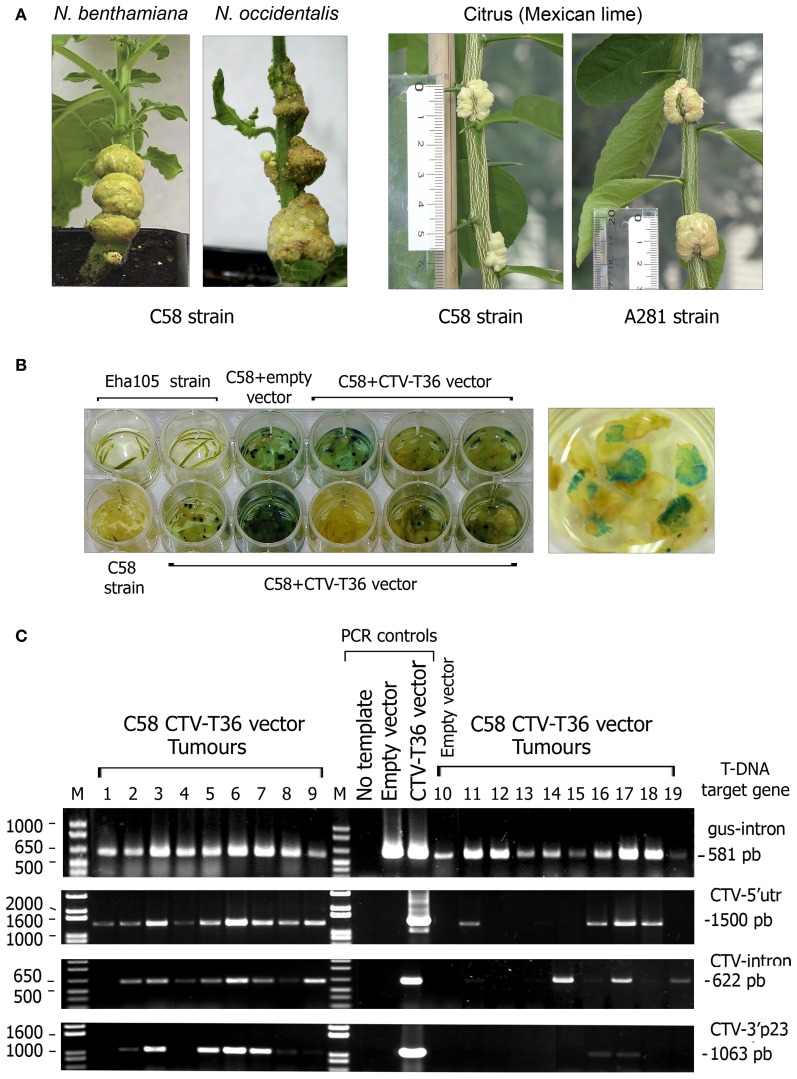
**Tumours incited by oncogenic *A. tumefaciens* strains in herbaceous and citrus hosts. (A)** Left panel, tumours induced by *A. tumefaciens* C58 strain in *Nicotiana* species at 4 weeks post inoculation (wpi) (NB) or 5 wpi (*N. occidentalis*). Right panel, tumours induced by *A. tumefaciens* C58 and A281 strains in Mexican lime at 7 wpi. The rule on the left indicates the size of individual tumours. **(B)** GUS activity assay of stem tumours from individual lime plants agroinoculated with *A. tumefaciens* C58. Each dish contains slices from individual tumours from plants agroinoculated with C58 harboring the pCH20-GUSi (empty vector) or the pCH20-GUSi-CTV (CTV-T36 vector) plasmids and incubated with X-Gluc solution at 8 wpi. Controls consisted of lime bark tissue agroinoculated with the non-oncogenic *A. tumefaciens* Eha105 strain or with the C58 strain without plasmid. A detail of tumor slices showing many blue spots of high-GUS activity is presented on the right. **(C)** Confirmation of partial T-DNA integrations in the cell nucleus of agroinfected lime plants. PCR amplification products obtained from gDNA extracts of tumours incited by *A. tumefaciens* C58 harboring the pCH20-GUSi-CTV or the pCH20-GUSi vectors (line 10). Controls: PCR amplification products from pCH20-GUSi-CTV (containing an intron in the CTV ORF 1a) and pCH20-GUSi plasmids, or from distilled water. The T-DNA target regions amplified (from the right to the left border as indicated in Figure 1) and the size of the DNA fragments are indicated at the right. Each lane corresponds to an individual tumor sample from different plants in the same experiment and DNA bands under the same number in different gels correspond to the amplification products obtained using different primers and the same gDNA (obtained from two slices of the same tumor). M, 1 Kb Plus DNA marker (Invitrogen, Fisher), with relevant sizes of DNA fragments indicated at the left.

In an attempt to simplify the newly developed genetic system for CTV (Ambrós et al., 2011) we tried direct agroinoculation of citrus with C58 and A281, two oncogenic strains of *A. tumefaciens* that produce good tumours in citrus plants, transfected with a suitable binary vector carrying the cDNA of CTV-T36 isolate (Satyanarayana et al., 2001; Ambrós et al., 2011) and an appropriate plant selectable marker to monitor cell transformation. For this purpose we developed the plasmid pCH20-GUSi, with a *gus*-intron marker gene (Figures 1A,B) that ensures that positive GUS expression was derived from transformed plant cells and not from residual bacteria, and then the vector pCH20-GUSi-CTV containing the expression cassette of CTV-T36 (Figure 1C). The functionality of this latter vector was first tested in *N. benthamiana* using the non-oncogenic *A. tumefaciens* strain COR308 and the ratio of CTV systemically infected plants obtained was similar to that reported previously (Ambrós et al., 2011 and data not shown). Suspensions of *A. tumefaciens* C58 and A281 harboring the pCH20-GUSi-CTV or pCH20-GUSi were then agroinoculated on the stems of several plant species, some susceptible to CTV infection (citrus and NB), and others (*N. occidentalis* and tomato) that are natural hosts for *A. tumefaciens* but not CTV hosts. Tomato plants were a positive control for the ability of both oncogenic strains to induce tumours (essentially 100% of the inoculation points) and a virulence phenotype, with A281 inciting more necrosis than C58 (data not shown). Similar results were observed in NB and *N. occidentalis* (Figure 2A, left panel). In Mexican lime, tumor formation frequency with both strains was also about 85%, but A281 elicited larger tumours than C58 and these appeared earlier (Figure 2A, right panel).

Analysis of GUS activity in tumor tissues revealed significant differences between A281 and C58 strains in tomato (~90% vs. 60% of GUS expressing tumours), whereas these percentages were similar for both strains in Mexican lime (~80%) (Table 1, Figure 2B). However, C58 induced more than twice the number of high-GUS-activity cells incited by A281 in lime tumours (Figure 2B, right panel), indicating higher frequency of independent transformation events. In NB C58 also induced over 90% of tumours with a high number of GUS-positive cells (Table 1).

**Table 1:** **Efficiency of tumor and systemic CTV infections in *N. benthamiana*, *N. occidentalis*, tomato cv. Roma (*L. sculentum L.*) and Mexican lime plants stem agroinoculated with oncogenic *A. tumefaciens* strains harboring the pCH20-GUSi empty vector or the pCH20-GUSi-CTV vector carrying the CTV expression cassette (CTV9R)**.

			**Source**		
			**Tumors**		**Plants**
**Plant host**	**Vector**	**Strain^a^**	**GUS^b^ activity**	**Infectivity^c^**	**Infectivity^d^**
*N. benthamiana*	CTV9R	C58	35/37	25/37	4/37
	Empty	C58	9/10	0/10	0/10
	CTV9R+p19^e^	C58	7/7	6/7	3/7
	Empty+p19^e^	C58	2/2	0/2	0/2
*N. occidentalis*	CTV9R	C58	6/7	0/7	0/7
	Empty	C58	2/2	0/2	0/2
Tomato	CTV9R	C58	19/36	0/19	0/19
		A281	14/15	0/7	0/7
	Empty	C58	6/8	0/5	0/5
		A281	9/9	0/3	0/3
	–	C58	0/3	0/3	0/3
	–	A281	0/3	0/3	0/3
Citrus	CTV9R	C58	118/138	0/44	0/44
	CTV9R	A281	72/90	0/31	0/31
	Empty	C58	2/3	0/3	0/4
		A281	3/4	0/4	0/4
	–	C58	0/2	0/2	0/2
	–	A281	0/2	0/2	0/2
	–	Eha105	–	–	0/2

a Oncogenic (C58 and A281) and disarmed (Eha105) A. tumefaciens strains used for stem agroinoculation.

b No. of N. benthamiana (NB) or N. occidentalis plants with GUS positive tumours/No. of plants with tumours. In tomato and citrus plants the values represent the No. of individual tumours with GUS activity/No. of tumours assayed (obtained from 3–4 independent bioassays).

c No. of plants with CTV infected tumours detected by ELISA at 2 mpi or later/No. of plants with tumours.

d No. of CTV systemically infected plants/No. of stem agroinoculated plants. Infection in upper leaves was detected by ELISA at 2–3 mpi or later.

e Co-infiltration with a vector expressing the p19 silencing suppressor protein of TBSV.

−Indicates agroinoculations with bacteria strains carrying no vectors, absence of GUS activity or absence of infectivity.

The efficiency of T-DNA integration in tumor cells was assessed by PCR detection of different target regions of the T-DNA insert using gDNA from tumours as template. In tomato, PCR assays detected three different T-DNA regions including the *gus*-gene and the CTV cDNA cassette in about 40% of the GUS positive tumours developed by both *Agrobacterium* strains. In Mexican lime, PCR amplified at least two of these T-DNA targets from 80 % of the tumor samples, and the four T-DNA regions including three of the CTV cDNA (Figure 2C), from 50% of the tumours. These results suggest that integration of the full T-DNA in the nucleus of lime cells by both oncogenic strains is relatively frequent.

**Figure 3:**
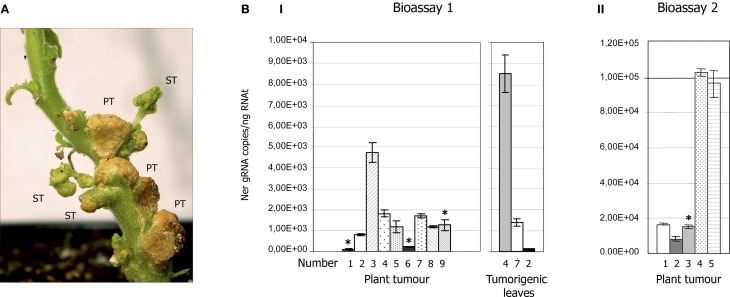
**CTV accumulation in tumours of NB agroinoculated with *A. tumefaciens* C58 harboring the pCH20-GUSi-CTV vector. (A)** Detail of tumor tissues induced by C58 in the stem of NB at 7 wpi showing three primary tumours (PT) and some tumorigenic leaves (ST, secondary tumours) that developed later beside the agroinoculation sites. **(B)** CTV accumulation in tumor tissues (or tumorigenic leaves) of NB plants agroinoculated with the C58 strain estimated by qRT-PCR (Ruiz-Ruiz et al., 2007) and expressed as number of CTV gRNA copies per ng of RNAt. Means and standard deviations were obtained from four technical replicates and two independent bioassays. Panels I and II show CTV accumulation in tumours of individual plants in bioassays 1 and 2, respectively. Asterisks above some bars indicate plants of these bioassays that became systemically infected by CTV. Right graphic in panel I shows the viral titer in tumorigenic leaves produced in three plants of bioassay 1.

Transient expression of the CTV cDNA was monitored by detecting the coat protein (ELISA) or the viral RNA (RT-PCR and qRT-PCR) in tumor tissues. While no positive CTV detection was ever observed in tomato tumours and only a faint amplification by qRT-PCR (with about 10^2^ CTV RNA copies/ng RNAt) was detected in some *N. occidentalis* galls, in NB most tumours became CTV infected as confirmed by positive ELISA readings, and the ratio of plants containing some CTV-infected tumor at 2 mpi was about 80% (Table 1). Accumulation of CTV gRNA in tumor tissues was very variable among plants of the same experiment and between assays, ranging from ~10^2^ to 10^5^ copies of CTV gRNA/ng RNAt (Figures 3BI,II). CTV expression was never observed in Mexican lime tumours, even when plants were co-inoculated with a binary vector expressing the p19 silencing suppressor of TBSV (Voinnet et al., 2003), suggesting either a strong plant silencing reaction against CTV or a failure to produce functional RNA transcripts in the lime tumor cells.

About 23% of the NB plants agroinoculated with the C58 strain transfected with pCH20-GUSi-CTV (with or without an intron in the CTV cDNA), became systemically infected by CTV at 2–3 mpi (Table 1), as confirmed by ELISA, qRT-PCR in upper leaves and by expression of specific symptoms. The number of transformation events obtained with C58 in NB seemed to increase when co-inoculated with a binary vector expressing the p19 silencing suppressor, as revealed by the slightly higher GUS activity and CTV systemic infection rate (Table 1). However, no association was observed between the presence of tumours with high CTV accumulation as detected by ELISA or qRT-PCR and systemic infection of the corresponding plant (Figures 3BI,II). Moreover, some plants with CTV-infected primary tumours developed new leaves beside the agroinoculation sites (Figure 3A) and these secondary tumorigenic tissues showed high CTV accumulation as confirmed by ELISA and q-RT-PCR (Figure 3BI, right panel), but systemic CTV infection did not occur.

**Figure 4:**
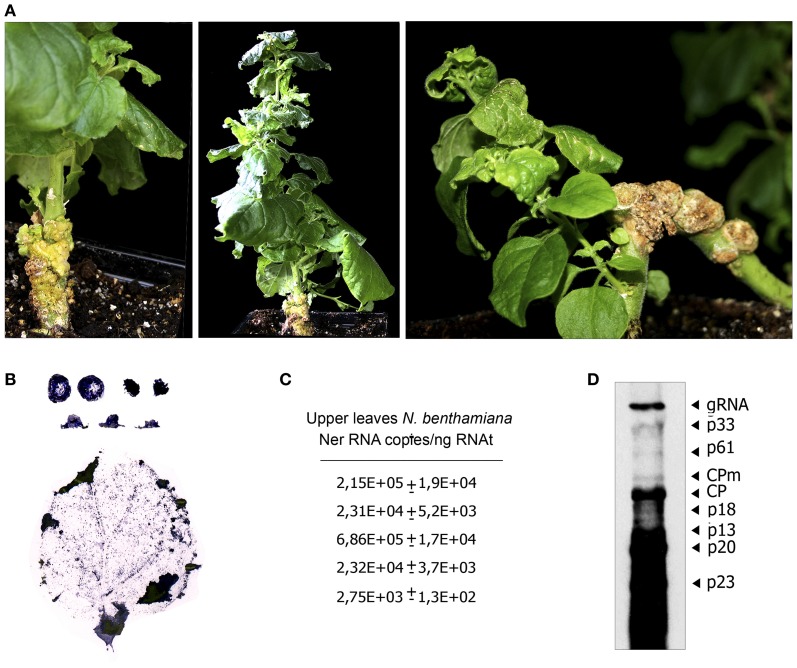
**Symptom expression and CTV distribution in NB plants systemically infected after agroinoculation with *A. tumefaciens* C58 harboring the pCH20-GUSi-CTV plasmid. (A)** Detail of symptoms induced by CTV in agroinfected *N*. *benthamiana*. Left, stem tumours and vein clearing symptoms in a new young lateral shoot (right) at 7 wpi. Middle, epinasty in NB at 8 wpi. Right, new shoot in an old systemically infected NB (10 wpi) showing stunting, vein clearing and crinkly leaf. **(B)** Relative accumulation and distribution of CTV in shoots (first line), petioles (second line) and upper leaves of systemically infected NB. **(C)** Absolute accumulation of CTV in upper leaves from different NB plants estimated by qRT-PCR (Ruiz-Ruiz et al., 2007) as in Figure 3. **(D)** Northern blot analysis of RNAt (4 μg) extracted at 10 wpi from the upper leaves of a CTV-infected NB plant. Positions of the CTV genomic (gRNA) and subgenomic RNAs (sgRNAs) are indicated by arrows on the right. The blot was hybridized with a digoxigenin-labeled riboprobe specific for the 3′-terminal region of the CTV-T36 gRNA.

CTV distribution as monitored by tissue-print-ELISA and symptom expression in systemically infected NB plants (Figures 4A,B) were similar to those reported using agroinfiltration with disarmed *Agrobacterium* strains (Ambrós et al., 2011). Viral titer in upper leaves was also variable among plants and experiments and generally ranged from ~10^4^ to 10^5^ CTV gRNA copies/ng RNAt (Figure 4C), albeit in some plants we observed a 2–60-fold excess of CTV gRNA in comparison with values obtained in plants agroinfiltrated with disarmed strains (Figures 4C,D, and Ambrós et al., 2011). Indeed when crude sap extracts from those plants were used to mechanically inoculate alemow plants, 42% of them (3/7) became infected. As expected, inoculation with purified virion preparations from the same NB plants resulted in 100% (4/4) of citrus plants infected at 1 mpi and they displayed the symptoms characteristic of the wild T36 isolate.

Systemic CTV infection was never observed in other agroinoculated hosts, as expected from low or no CTV expression in their tumours. Moreover, agroinfiltration of Etrog citron (*C. medica* L.), alemow or Mexican lime leaves with the C58 and A281 oncogenic strains, albeit inducing functional leaf tumours, did not result either in systemic infection and only a faint qRT-PCR amplification of CTV targets was occasionally observed at 14–28 dpi, suggesting restricted infections that never progressed.

### Pre-treatment with a silencing suppressor enabled systemic infection of NB plants after mechanical inoculation with CTV virions

Previous attempts to mechanically transmit CTV from citrus to herbaceous or non-rutaceous woody species, including NB, were unsuccessful (Müller and Garnsey, 1984 and our unpublished results). Since experimental aphid transmission of CTV to some *Passiflora* species has been reported (Müller et al., 1974; Roistacher and Bar-Joseph, 1987), we tried to aphid transmit CTV-T36 to healthy NB plants using *A. gosypii* (>100 individuals) fed for 24 h on infected citrus or NB leaves. Although CTV was detected in some aphids by qRT-PCR no receptor plant was infected.

We then examined other inoculation procedures that could enable testing infectivity of different CTV genotypes in NB without using protoplasts or aphids. For this purpose leaves of young NB plants were mechanically inoculated with crude sap from systemically infected (T36 strain) NB leaves or with purified CTV virions from these plants or from CTV-infected citrus bark (Table 2). Since all transmission trials were unsuccessful, bioassays were repeated agroinfiltrating the receptor plants with a vector expressing the p19 silencing suppressor (TBSV) 3 days before mechanical inoculation. Unexpectedly, using this pre-treatment we were able to mechanically transmit for the first time CTV virions to NB (Table 2). Although transmission efficiency was variable among bioassays, an average of 27–39% infected plants was obtained, with vein clearing and stunting symptoms being indistinguishable from those previously observed in this host (Ambrós et al., 2011), albeit they appeared at 3–4 mpi (a 2–3 months delay in comparison with the leaf agroinfiltration procedure).

**Table 2:** **Transmission of CTV by mechanical inoculation of NB healthy plants with or without pre-treatment with a silencing suppressor**.

**CTV isolate**	**Inoculum source**	**Type of inoculum^a^**	**Suppressor treatment^b^**	**Infectivity^c^**
T36	Infected NB upper leaves^d^	Crude sap	-	0/6, 0/6
	Infected NB upper leaves	Virions	-	0/6, 0/6
	Infected citrus bark	Virions	-	0/6
	Infected NB upper leaves	Crude sap	p19	0/6, 0/6
	Infected NB upper leaves	Virions	p19	2/3, 0/3, 2/4
	Infected citrus bark	Virions	p19	2/4, 1/3, 0/3
T318A	Infected citrus bark	Virions	-	0/6
	Infected citrus bark	Virions	p19	0/3, 0/3

a Crude sap extracts or gradient purified virions.

b Pre-infiltration with a vector expressing the p19 silencing suppressor protein of TBSV.

c No. of infected NB plants/No. of inoculated plants at 2–3 mpi or later in independent bioassays.

d Upper leaves from systemically infected NB plants.

**Figure 5:**
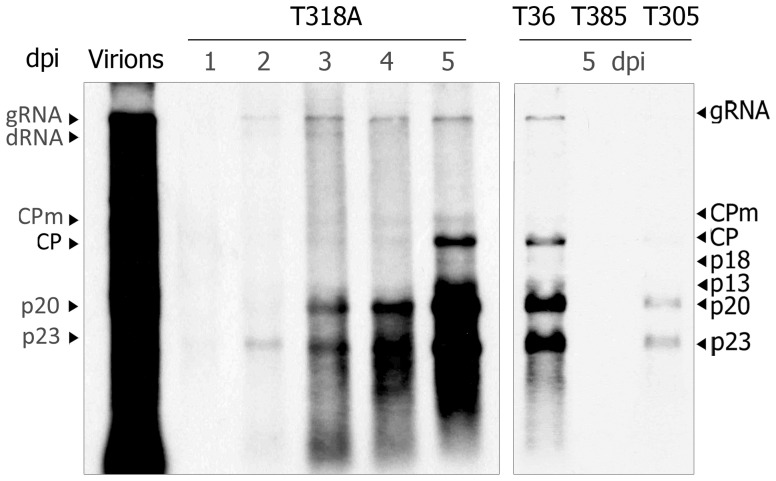
**Northern blot analysis of RNAt from NB mesophyll protoplasts transfected with purified virions from citrus plants infected with the CTV isolate T318A at 1 through 5 days post-inoculation (dpi) (left panel), and the T36, T385, and T305 isolates at 5 dpi (right panel)**. Left lane, virion extract used to transfect protoplasts. Positions of the CTV gRNA and sgRNAs are indicated by arrows at both sides. A large dRNA present in T318A and its relative position is shown on the left. The blot was hybridized with a digoxigenin-labeled riboprobe specific for the 3′-terminal region of the CTV-T318A gRNA.

Since not all CTV genotypes are able to successfully replicate in NB (Navas-Castillo et al., 1997; Satyanarayana et al., 2000) we first assayed virion infectivity of T318A and T305, two Spanish isolates inducing stem pitting in grapefruit and sweet orange, in NB protoplasts in comparison with T36 and T385 virions, used as positive and negative controls, respectively. Northern blot analysis of protoplasts transfected with T318A virions showed viral progeny accumulation at 2 dpi with the viral gRNA and sgRNAs being readily visible at 3 dpi (Figure 5, left panel). Generally, viral RNA accumulation of this isolate was similar to that of the CTV-T36 control, and higher than that displayed by T305. Moreover, accumulation of T318A RNAs increased up to 5 dpi in surviving protoplasts and their hybridization signal remained as intense as that of CTV-T36, whereas this signal was very weak for T305, and no signal was observed for T385 (Figure 5, right panel). Although T318A virions successfully replicated in NB protoplasts, mechanical inoculation of these virions on NB plants, with or without an agroinfiltration pre-treatment with the p19 silencing suppressor, failed to cause systemic infection of those plants (Table 2).

Overall, these results indicate that the interaction between CTV-T36 and NB must be genotype-specific.

### CTV can be graft-transmitted from infected to healthy NB plants

**Figure 6:**
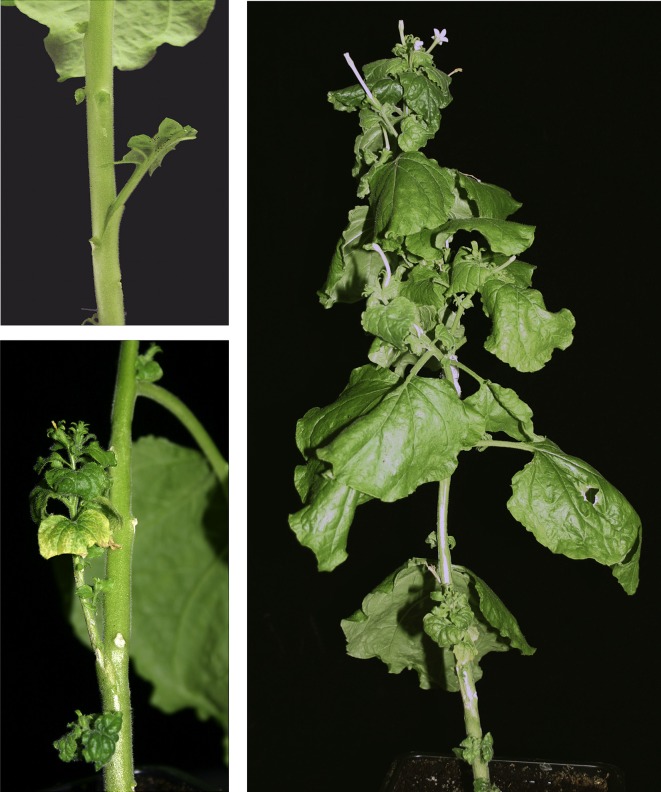
**CTV systemic infection of NB plants after side-graft inoculation**. Left, detail of side-graft inoculation of NB plants using CTV infected petioles (upper panel) or young shoots with vein clearing symptoms (lower panel). Right, severe stunting and crinkly leaf in a NB plant systemically infected (7 wpi); one surviving graft shows CTV symptoms.

To maintain wild or mutant CTV genotypes in NB without starting periodically a new agroinfection process we tried to develop a system to graft-transmit CTV from systemically infected to healthy NB plants. For this purpose we assayed different types of tissues from the donor plant putting two side-grafts per receptor plant and protecting them to avoid desiccation. Suculent petioles or short young shoots (Figure 6) from CTV-infected NB plants were the best inoculum source since larger shoots or stems showed reduced survival. Around 40% graft survival was observed at the end of the first month, with surviving grafts showing CTV symptoms or fluorescence when a *gfp*-tagged virus (Tatineni et al., 2008) was used (Figure 6). In spite of inoculum survival, essentially no CTV transmission was observed in receptor NB plants without pre-treatment with a silencing suppressor, except for a single plant out of the 70 inoculated in several experiments (Table 3). In contrast, agroinfiltration with binary plasmids expressing the p19 (TBSV) or the p23 (CTV) silencing suppressors 3 or 6 days prior to graft inoculation resulted in 50 or 28% average transmission rates, respectively, at 2 mpi. The rate of infected plants still increased at 4 mpi for plants pre-treated with p19.

**Table 3:** **Side-graft transmission of CTV from infected to healthy NB plants with or without pre-treatment with a silencing suppressor**.

**Silencing suppressor^a^**	**Pre-treatment day^b^**	**Infectivity^c^**	**%^d^**
-	-	0/17, 0/13, 0/21, 1/19	<2%
p23 p23	D3 D6	2/9 3/9	22% 33%
p19 p19	D3 D6	8/9, 3/5, 3/5 5/9, 8/17	70% 52%

a Pre-infiltration with a vector expressing the p19 silencing suppressor protein of TBSV or the p23 silencing suppressor protein of CTV.

b Pre-infiltration of leaves of the receptor plants was performed 3 or 6 days prior to graft inoculations.

c No. of infected NB plants/No. of inoculated plants in independent bioassays at 4–6 mpi.

d Average transmission rate.

Symptoms of infected plants were similar to those reported before, but they appeared earlier than in plants inoculated mechanically, since vascular connections between the graft and the receptor plant likely allowed direct loading of CTV virions into the phloem tubes.

## Discussion

A new genetic system based on agroinfiltration of NB leaves with disarmed *Agrobacterium* cultures transfected with binary vectors, carrying the CTV-T36 cDNA, was recently developed (Ambrós et al., 2011). Although this system was easier, faster and more reliable than the former protoplast system (Satyanarayana et al., 1999, 2001), it still showed limitations that we tried to overcome in this work. The most obvious was, why can we not directly agroinoculate citrus plants? *A. tumefaciens* has been widely used for efficient delivery of viral genomes into different plants including a citrus-infecting virus and several phloem-limited viruses some of which belong to the *Closteroviridae* family (Grimsley et al., 1986; Prokhnevsky et al., 2002; Chiba et al., 2006; Vives et al., 2008; Liu et al., 2009; Wang et al., 2009). Although transient or stable transformation of citrus species has also been demonstrated (Cervera et al., 1998; Tzfira et al., 2004; reviewed in Peña et al., 2004a), previous trials to agroinfect citrus plants with CTV using different binary plasmids, silencing suppressors and disarmed *Agrobacterium* strains were unsuccessful (Gowda et al., 2005, and our unpublished data). Infiltration of citrus leaves with *Agrobacterium* is usually very inefficient for bacterial penetration and usually leads to low level of T-DNA expression, as observed with other recalcitrant species (Wroblewski et al., 2005). However, the ability of wild oncogenic strains to incite tumours on some citrus species proved very efficient for citrus transformation (Cervera et al., 1998). Here the virulent strain C58 was as efficient as the supervirulent strain A281 inducing tumor formation in Mexican lime plants, albeit tumours induced by the latter grew earlier, were larger in size and showed a necrotic phenotype, in agreement with the supervirulence reported by Cervera et al., (1998). Both oncogenic strains, carrying the empty or the CTV expressing vector, produced good vascularized tumours and high GUS transient expression in NB and lime, with C58 giving better results than A281 in the latter host species. Using a *gus*-intron marker gene engineered in the T-DNA of the binary vector guaranteed that GUS expression occurred only *in planta* and, therefore, it was indicative of cell transformation. Most tumours incited by C58 in NB became CTV infected at 1–2 mpi, as detected by ELISA and qRT-PCR assays, and 23% of the plants became systemically infected by CTV at 2–3 mpi, as expected from the high vascularization of these tumours. Remarkably, systemic infection of these plants was not associated with highest CTV titers in tumours. Moreover, some plants developed secondary tumorigenic tissues with high CTV titer, but systemic infection of these plants never occurred, further supporting our previous suggestion that systemic infection of NB results from CTV invasion of the vascular system and not from *Agrobacterium* migration within agroinfiltrated plants (Cubero et al., 2006; Ambrós et al., 2011). Contrasting with NB, essentially no CTV coat protein or viral RNA was detected in *N. occidentalis* tumours in spite of the high GUS expression observed, suggesting limited virus replication if any in cells of this host, in agreement with the lack of CTV replication observed previously in protoplasts of this species (Navas-Castillo et al., 1997; Satyanarayana et al., 2000). Neither CTV coat protein nor viral RNA was detected in Mexican lime tumours in spite of the high proportion of cells showing intense GUS activity, indicative of total or partial T-DNA integration. Moreover, PCR detection of different T-DNA regions in individual tumours suggested that integration of the full CTV cDNA in the Mexican lime cells occurs at an effective rate. Therefore, failure to detect CTV infection in lime tumours does not seem to depend on the oncogenic strain, tumor formation or cell transformation efficiency. Indeed co-agroinoculation with a binary plasmid expressing the p19 silencing suppressor (Voinnet et al., 2003) slightly increased GUS activity and the CTV systemic infection rate in NB tumours, but it had no effect in lime tumours. Since Mexican lime is known to be highly susceptible to CTV infection, our results suggest a strong silencing response against CTV at the very early steps of infection, or more likely, failure to get enough functional RNA transcripts reaching the cytoplasm of transformed lime cells. Moreover, alemow and Mexican lime leaves pre-infiltrated with a silencing suppressor and then with the vector carrying CTV-T36 developed tumours, but only trace amounts of CTV gRNA could be detected by qRT-PCR in some of them at 14–28 dpi, suggesting occasional restricted infections that never progressed. These results indicate that a CTV genetic system based on direct agroinoculation of citrus hosts presently is unworkable and that, at least in the near future, the use of NB as an intermediate host to produce CTV virions will be necessary.

Since the present genetic system relies on the ability of CTV-36 to replicate in NB cells and to eventually move cell-to-cell and long distance, developing a similar system with new CTV genotypes requires testing previously the ability of their virions to replicate in NB cells. Previous observations indicated that not all CTV genotypes can replicate in NB protoplasts. Here we confirmed that while isolate T318A (Ruiz-Ruiz et al., 2006) replicated and accumulated in protoplasts to the same extent as CTV-T36, the isolate T305 replicated at low level and the isolate T385 did not replicate at all. Phylogenetic comparison of the full-genome sequence of different CTV isolates has revealed that these belong to at least six different strains, with most genetic differences being located in the 5′ moiety of the gRNA (Harper, 2013). Differences in the replicase components may affect their interactions with host factors and thus determine the ability of each strain to replicate in NB protoplasts. To avoid the need for protoplast preparation we tried to mechanically inoculate NB plants by rubbing leaves with CTV-T36 and T318A virion extracts from citrus. While no infection was observed in plants without pre-treatment with a silencing suppressor, up to 39% infection rate was achieved in pre-treated plants mechanically inoculated with CTV-T36 virions. This is the first time that CTV is mechanically transmitted to a new host by leaf rubbing. Although co-infiltration of NB plants with a silencing suppressor was not essential for CTV agroinfection with disarmed *A. tumefaciens* strains, in line with results reported for the crinivirus *Lettuce infectious yellowing* (LIYV) (Wang et al., 2009), silencing suppressors expedited systemic infection and often increased infectivity (Ambrós et al., 2011). This finding and the need for a silencing-suppressed receptor plant to achieve infection in mechanically inoculated plants underlines the importance of the antiviral silencing reaction in the early stages of CTV infection. Unfortunately no infection was obtained in pre-treated plants inoculated with T318A virions, even though this genotype replicated like CTV-T36 in NB protoplasts, suggesting that viral factors other than the replicase interact differently with host factors in T36 and in T318 genotypes. Previous studies have shown that resistance to CTV infection is often strain dependent (Yoshida, 1985, 1993, 1996; Garnsey et al., 1987; Gmitter et al., 1996; Mestre et al., 1997a,b,c). Furthermore, this resistance was often due to inability of the virus to move cell-to-cell or long distance, since CTV replicated and accumulated in protoplasts of resistant citrus varieties to the same extent as in susceptible varieties, and normal infectious virions were produced (Albiach-Martí et al., 2004). It is possible that, although the CTV T318A gRNA efficiently replicates in *N. benthamiana* protoplasts and likely in cells, virions may not be correctly assembled, or virion proteins may not interact properly with host factors, thus impairing virus movement. These results are a major concern for the actual possibilities to use the present genetic system with CTV genotypes other than CTV-T36. Additional CTV isolates replicating in NB protoplasts should be tested for infectivity on NB plants to ascertain if systemic infection of this species is T36-specific or it can be achieved by other virus genotypes. In any case, availability of CTV genotypes capable to produce systemic infection of this host species, others able to replicate but not to spread systemically, and still others unable to replicate, may be helpful to dissect the CTV-NB interactions at the genetic level.

A final limitation of the new genetic system based on agroinoculation of NB plants was the need for new plant agroinoculation cycles to maintain in this host the CTV-T36 or other interesting hybrid constructs that could be produced. This limitation was overcome by developing an efficient graft inoculation system to transmit CTV from infected to healthy NB plants. Pre-treatment of the receptor plants by agroinfiltrating a silencing suppressor, proper selection of the inoculum for long-term survival and incubation conditions to avoid inoculum desiccation were critical factors that increased the transmission rates. Although side grafts allow direct contact of the donor and the receptor phloem tissues, pre-treatment with a silencing suppressor raised transmission rate from less than 2% to about 50% at 3 mpi, underlining again the importance of antiviral silencing defense of the plant in the early stages of CTV infection. Several reports have documented that virus loading into the vascular system is a complex process involving a strong bottleneck for the virus population (Ding et al., 1995; Gilbertson and Lucas, 1996; Wintermantel et al., 1997; Cruz, 1999; Li and Roossinck, 2004; Ali and Roossinck, 2010). Reduction in the effective population size and virus silencing by the new host might explain in part the long delay necessary for systemic infection. Finding that higher infectivity was obtained pre-treating with p19, a silencing suppressor acting at cellular and systemic levels (Voinnet et al., 2003), than with p23, a CTV-encoded suppressor acting only at cellular level (Lu et al., 2004), support the idea that virus loading in the vascular system is an important step for systemic infection and that suppressing long-distance silencing of the receptor plant helps to overcome this obstacle. Moreover, plants pre-treated with p19 not only showed a higher rate of infection at 2 mpi but, contrasting with those pre-treated with p23, this rate further increased at 4 mpi.

CTV distribution and symptom expression in NB plants systemically infected after agroinoculation with oncogenic strains, mechanical inoculation of virions or graft transmission, mimic those reported previously on this host (Ambrós et al., 2011), supporting the notion that essential host-viral interactions leading to viral movement and symptom development remain unaltered. The main difference between agroinoculation and mechanical or graft inoculation was the significant delay in systemic CTV infection observed with the two latter methods, particularly with mechanical inoculation that sometimes had a lag period of up to 4–6 months, probably due to the low number of CTV virions initiating infection. Biological characteristics of CTV virions from NB systemically infected by either procedure remain unaltered and upon slash inoculation to alemow plants these displayed the symptoms characteristic of the CTV-T36 isolate.

Summarizing, agroinoculation of NB with CTV-T36-based vectors by either agroinfiltration or stem agroinoculation are presently the best procedure to assay new CTV hybrid constructs in citrus plants. These new constructs can now be easily maintained in NB plants for future transmissions to citrus or for eventual studies on CTV stability or evolutionary adaption to this non-natural host after successive passages. Infection of NB by leaf rubbing with virion extracts is to our knowledge the first report on how a non-natural host species may become susceptible to mechanical inoculation with a virus that is naturally phloem-restricted, after pre-treatment with a silencing suppressor. This procedure may potentially help developing future infectious CTV clones if virus genotypes other than CTV-T36 replicating in protoplasts are found capable of invading NB plants.

### Conflict of interest statement

The authors declare that the research was conducted in the absence of any commercial or financial relationships that could be construed as a potential conflict of interest.
